# The Practical Landscape of Cytokine‐Targeted miRNAs to Enhance NK Cell Function in Cancer Immunotherapy: A Bioinformatic Analysis

**DOI:** 10.1002/cnr2.70192

**Published:** 2025-04-06

**Authors:** Arefeh Zabeti Touchaei, Sogand Vahidi, Ali Akbar Samadani

**Affiliations:** ^1^ Department of Chemistry Lahijan Branch, Islamic Azad University Lahijan Iran; ^2^ Medical Biology Research Center Kermanshah University of Medical Sciences Kermanshah Iran; ^3^ Guilan Road Trauma Research Center, Trauma Institute Guilan University of Medical Sciences Rasht Iran

**Keywords:** Cancer immunotherapy, cytokines, miRNAs, natural killer (NK) cells, tumor microenvironment

## Abstract

**Introduction:**

Suppression within the tumor microenvironment (TME) hampered natural killer (NK) cells and their role in cancer immunotherapy. This study explores how interleukin (IL) signaling (IL‐12A, IL‐12B, IL‐15, IL‐18) and interferon gamma (IFNG or IFN‐γ) interact with microRNAs to regulate NK cell function in cancer.

**Methods:**

We identify the targeted microRNAs (miRNAs) for these genes and the key pathways influencing various cancers through comprehensive analyses, including protein–protein interaction networks, protein co‐expression, miRNA targeting prediction, homology, mRNA‐miRNA regulatory networks, gene set enrichment, and signaling pathway analysis.

**Results:**

Our analysis revealed a significant association between genes encoding interleukins and IFNG with NK cell infiltration across various cancers. Additionally, we identified several miRNAs (hsa‐miR‐590‐3p, hsa‐miR‐340‐5p, hsa‐miR‐495‐3p, hsa‐miR‐5692a, hsa‐miR‐130a‐3p) that potentially regulate NK cell function by targeting these genes. These miRNAs participate in critical pathways essential for NK cell function. Notably, our findings suggest a key role for mRNA‐miRNA co‐regulation in suppressing NK cells within the tumor microenvironment.

**Conclusion:**

This study highlights the potential of targeting these identified miRNAs as a strategy to enhance NK cell function and improve the efficacy of cancer immunotherapy.

**Abbreviations**
ADCC: antibody‐dependent cellular cytotoxicity
BP: biological process
CC: cellular component
DAMPs: damage‐associated molecular patterns
GO: gene ontology
GM‐CSF: granulocyte‐macrophage colony‐stimulating factor
IFNs: interferons
IL: interleukin
IFNG or IFN‐γ: interferon‐gamma
JAK‐STAT: Janus kinase/signal transducers and activators of transcription
KEGG: Kyoto encyclopedia of genes and genomes
MAPKs: mitogen‐activated protein kinases
MF: molecular function
MFE: minimum free energy
miRNAs: microRNAs
NK: natural killer cells
NF‐κB: nuclear factor kappa B
PPIs: protein‐protein interactions
STAT4: signal transducer and activator of transcription 4
Th: T helper
TIMER 2.0: Tumor Immune Estimation Resource 2.0
TLRs: toll‐like receptors
TME: tumor microenvironment
TNF‐α: tumor necrosis factor‐α
UTR: untranslated region

## 1 Introduction

Natural killer (NK) cells function by generating cytokines and chemokines in response to external factors, including the cytokines themselves and activating receptors on their surface. Cytokines are essential in shaping every stage of an NK cell's life, from expansion and activation to ensuring its survival. Their impact is both profound and crucial. While some cytokines, like interleukin (IL)‐23 and IL‐27, can have context‐dependent effects, a specific group–including IL‐2, IL‐12, IL‐15, IL‐18, IL‐21, and type I interferons (IFNs)–consistently promotes NK cell function, either individually or in combination. In contrast, cytokines like TGFβ, IL‐6, and IL‐10 act as immunosuppressors within the tumor microenvironment (TME). They directly inhibit NK cell activity and indirectly create a suppressive environment by influencing other immune cells and counteracting the effects of stimulatory cytokines [1, 2]. This dampens the ability of NK cells to fight tumors and facilitates tumor evasion and growth. interferon‐gamma (IFNG or IFN‐γ) is a potent effector cytokine produced by NK cells; it has pleiotropic effects on target cells that are malignant or infected with viruses, as well as on other immune and antigen‐presenting cells [3, 4].

IL‐12 triggers IFNG production via signal transducer and activator of transcription 4 (STAT4) phosphorylation and T‐bet activation [5]. IL‐15 plays a critical duty in NK cell biology, promoting survival, proliferation, differentiation, and activation via STAT5 phosphorylation upon receptor binding [6, 7, 8]. IL‐18, as a member of the type 1 cytokine family, synergizes with IL‐12 to induce high levels of IFNG in human NK cells. This synergy involves IL‐18 activating the p38 mitogen‐activated protein kinases (MAPKs) and nuclear factor kappa B (NF‐κB) pathways, which enhance the stability of IFNG transcripts. Additionally, combined IL‐18 and IL‐12 stimulation leads to the release of granulocyte‐macrophage colony‐stimulating factor (GM‐CSF) and tumor necrosis factor‐α (TNF‐α) [9, 10].

NK cells are promising candidates for cancer immunotherapy because of their role in immune surveillance. However, these cells often become dysfunctional in cancer patients. This dysfunction is most pronounced within the TME of advanced malignancies, but it also extends to NK cells circulating in the blood and residing in other organs [11, 12].

MicroRNAs (miRNAs) are known to be influenced by both innate and adaptive immune responses. Recent research highlights their critical role in NK cell function, although novel findings in this area are not widely disseminated. Interestingly, miRNAs can function as either oncogenes or tumor suppressors in various cancers. Notably, the majority of these endogenous miRNAs appear to regulate NK cell antitumor activity within the TME [13].

NK cell activation is crucial for their anti‐tumor function. Inhibitory receptors on the NK cell surface can prevent this activation by blocking essential signaling pathways. Specifically, they suppress intracellular calcium signaling and reduce the phosphorylation of critical NK cell components. Conversely, activating receptors trigger downstream signaling through their tyrosine activating motifs upon ligand binding. This activation pathway leads to an increased release of cytotoxic molecules like IFNG, perforin, and granzyme B, enabling NK cells to eliminate tumor cells [14].

NK cells eliminate target cells through a multi‐pronged attack. They primarily deploy perforin and granzymes alongside IFNG. While recent research highlights the critical role of lncRNAs in regulating IFNG secretion, perforin and granzyme B expression are primarily controlled by miRNAs. These miRNAs can enhance NK cell cytotoxicity by increasing the release of these cytotoxic molecules. They achieve this by targeting tumor suppressor proteins or directly binding to the 3' untranslated region (3'‐UTR) of perforin and granzyme B mRNA, regulating their expression levels [15].

NK cells, known for their potent and rapid anti‐tumor activity, hold promise for cancer immunotherapy. However, progress has been hampered by challenges in large‐scale NK cell collection, ex vivo expansion, and maintaining their survival and function in vivo. Despite these hurdles, recent advancements in prediction techniques and NK cell biology offer renewed opportunities to explore the therapeutic potential of NK cells through novel approaches [16].

This study aimed to elucidate the interplay between IL‐12A, IL‐12B, IL‐15, IL‐18, and IFNG genes in NK cell function across various cancers. Additionally, we sought to identify and predict the miRNA targets of these genes, exploring their potential role in regulating NK cell activity and signaling pathways. Ultimately, this investigation could pave the way for unknown therapeutic approaches involving NK cell modulation through targeted miRNAs for improved cancer treatment and immunotherapy.

## 2 Materials and Methods

### 2.1 KEGG Pathway and PPI Network Analysis

We utilized the STRING database (https://string‐db.org) to create a protein interaction network for IL‐12A, IL‐12B, IL‐15, IL‐18, and IFNG. This database combines known and predicted protein–protein interactions (PPIs) with protein co‐expression data. Additionally, we employed the KEGG pathway database to generate a network of functionally enriched pathways associated with these genes.

### 2.2 Immune Infiltration Analysis

To explore the relationship between the expression of IL‐12A, IL‐12B, IL‐15, IL‐18, and IFNG genes and immune cell infiltration (specifically NK cells), we utilized the Tumor Immune Estimation Resource 2.0 (TIMER 2.0) database (http://timer.cistrome.org) accessed in July 2023. Within the “Immune‐Gene” module, we analyzed the infiltration of NK cells by setting “IL‐12A”, “IL‐12B”, “IL‐15”, “IL‐18”, and “IFNG” as gene expression keywords. Only correlations with a Spearman's *p*‐value of less than 0.05 were considered for further analysis.

### 2.3 Prediction of Target Regulatory miRNAs for Genes IL‐12A, IL‐12B, IL‐15, IL‐18, and IFNG

We employed a combination of miRNA target prediction databases to identify potential miRNA regulators of IL‐12A, IL‐12B, IL‐15, IL‐18, and IFNG genes. These databases included TargetScan, miRWalk, miRTarBase, miRTar2GO, miRDB, and miRSystem, which encompass resources like DIANA, miRanda, miRBridge, PicTar, PITA, and RNA22.

### 2.4 miRNA and Target Sequence Homology

We retrieved the 3′ untranslated region (UTR) sequences of the investigated genes (IL‐12A, IL‐12B, IL‐15, IL‐18, and IFNG) from the NCBI database to identify potential miRNA binding sites. Mature miRNA sequences were obtained from the miRDB database (https://mirdb.org), which uses the MirTarget algorithm to predict miRNA targets based on high‐throughput sequencing data and machine learning methods. Homology analysis between the gene 3' UTR sequences and the mature miRNAs was conducted using the BiBiserv RNA hybridization tool [17]. For this analysis, a minimum free energy (MFE) value of −15 kcal/mol was used as the threshold for significant miRNA–mRNA interactions, with stronger interactions defined by an MFE of ≤ −30 kcal/mol, indicating a more stable duplex formation.

### 2.5 miRNA Co‐Regulatory Network

Based on the predicted interactions between miRNAs and their target genes, we identified miRNA‐miRNA regulatory relationships. The miRNA‐mRNA interaction network was visualized utilizing Cytoscape software version 3.10.0 [18].

### 2.6 Functional and Pathway Enrichment Analysis

We utilized two approaches to further investigate the functional roles of identified miRNAs and hub genes. First, we utilized DIANA‐miRPath v3.0 (https://dianalab.e‐ce.uth.gr/html/mirpathv3/index.php?r=mirpath) (accessed in July 2023) to perform pathway analysis. This tool identified relevant Kyoto Encyclopedia of Genes and Genomes (KEGG) pathways associated with the miRNAs and their target genes. Second, we leveraged the “clusterProfiler” package from the Bioconductor software suite in R for gene ontology (GO) analysis. This analysis categorized the genes based on their biological process (BP), cellular component (CC), and molecular function (MF), further elucidating their roles within the cell. To avoid reporting terms lacking biological or statistical significance, GO terms represented by fewer than two genes were excluded from further analysis. A significance threshold of *p*‐value < 0.05 was applied [19]. To visualize the relationship between miRNAs and enriched pathways, we utilized the heatmap function within DIANA‐miRPath v3.0. The heatmap was generated using the pathway intersection mode, which highlights pathways commonly targeted by multiple miRNAs. The hierarchical clustering applied to both axes groups miRNAs and pathways based on their enrichment patterns.

## 3 Results

### 3.1 Enrichment Analysis

**FIGURE 1:**
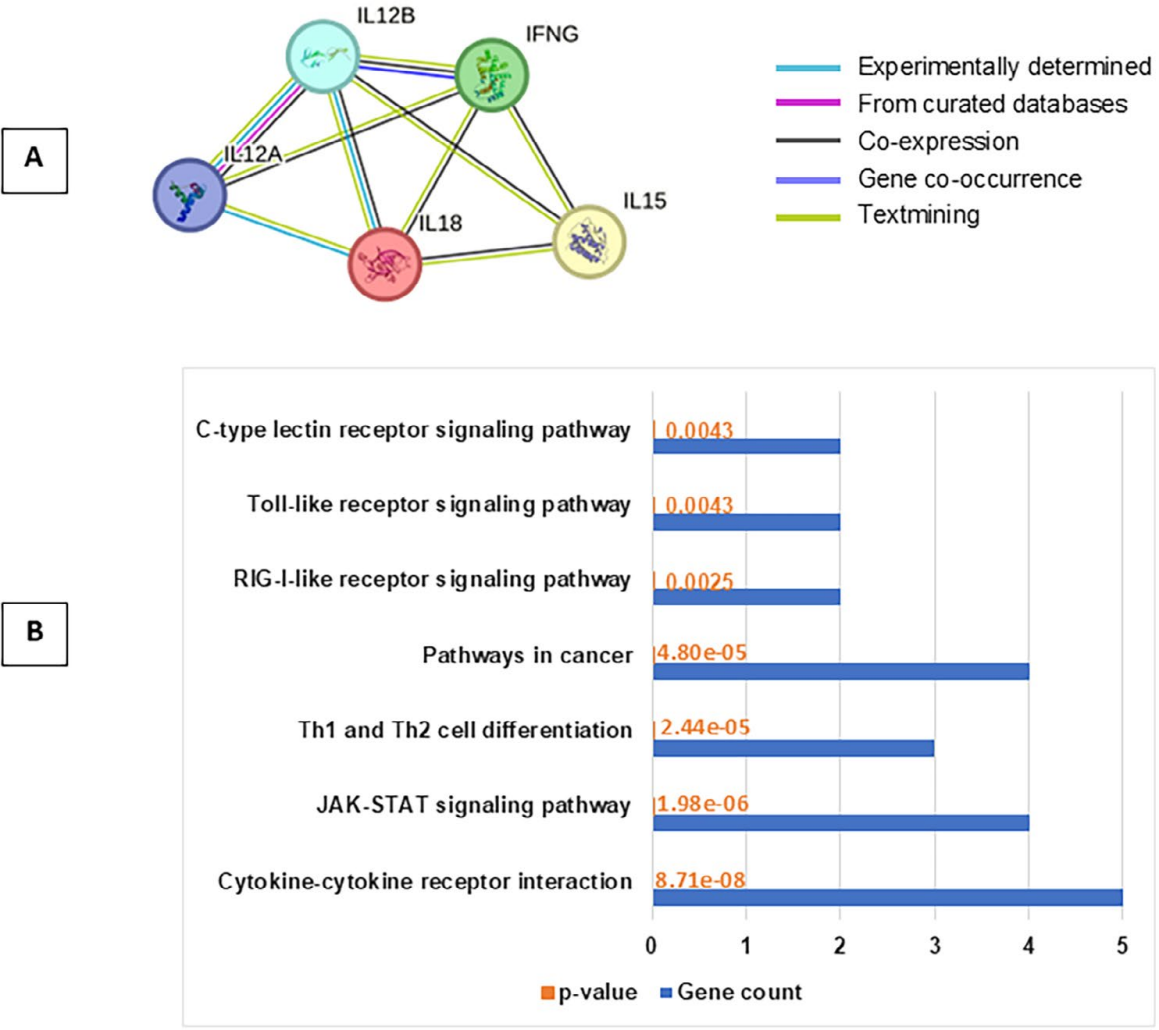
PPI and KEGG analyze IL‐12A, IL‐12B, IL‐15, IL‐18, and IFNG genes. (A) Protein–protein interaction network of genes. Spheres represent the nodes and the lines represent the interactions. (B) KEGG pathway enrichment analysis. (C) Pathway enrichment analysis of IL‐12A, IL‐12B, IL‐15, IL‐18, and IFNG genes.

Figure 1A depicts the protein–protein interaction (PPI) network of the investigated genes. This network demonstrates a significantly higher number of interactions than expected by chance, suggesting a high degree of biological connectivity among these proteins (*p*‐value: 4.3e‐07). The network consists of 5 nodes linked by 9 edges (proteins linked by interactions, respectively).

Co‐expression analysis in humans further supports this functional connection (Figure 1B). The color intensity in the figure represents the level of confidence in the functional association between protein pairs. Notably, all five genes (IL‐12A, IL‐12B, IL‐15, IL‐18, and IFNG) exhibit co‐expression, suggesting their coordinated roles in biological processes.

Finally, pathway analysis using the KEGG database (Figure 1C) revealed enrichment in pathways critical for malignancy and tumor treatment, including cytokine‐cytokine receptor interaction, Janus kinase/signal transducers and activators of transcription (JAK–STAT) signaling, pathways in cancer, and T helper (Th), Th1/Th2 cell differentiation.

### 3.2 Relation Between Immune Cell Infiltration and Gene Expression in Multiple Types of Cancer

**FIGURE 2:**
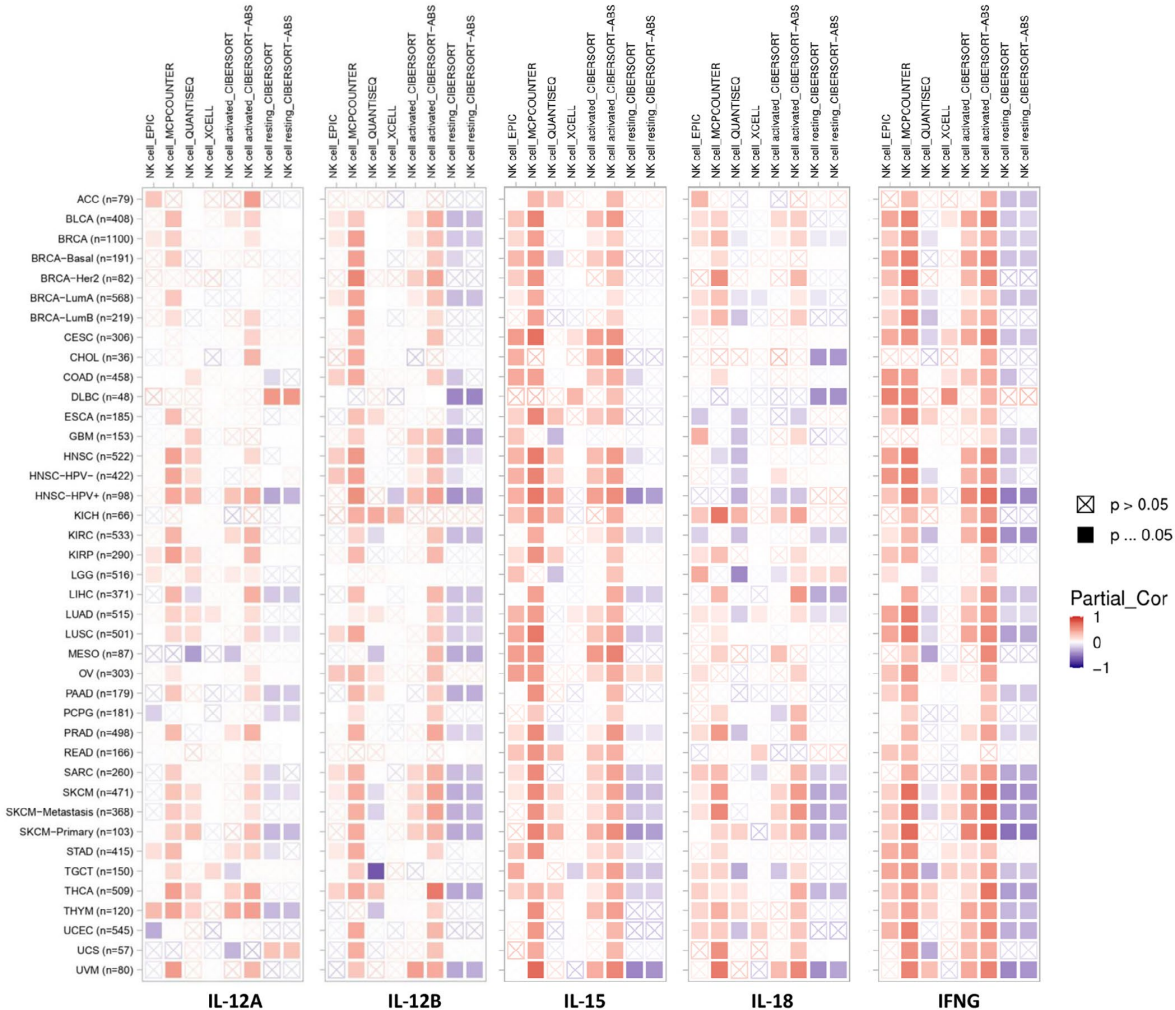
Correlation between IL‐12A, IL‐12B, IL‐15, IL‐18, and IFNG expression and immune cell infiltration in various cancer types. Correlations between the above genes expression and infiltration of NK cells were analyzed using TIMER 2.0. To determine significance, Spearman's correlation analysis was used. Positive and negative correlations are represented by red and blue blocks, respectively. *p* < 0.05 was considered significant.

Building on the link between IL‐12A/B, IL‐15, IL‐18, and IFNG expression and immune pathways, we investigated their correlation with NK cell infiltration in various cancers (Figure 2). Spearman's correlation analysis revealed a positive association between these genes' expression and NK cell levels across cancer types, supporting the expected link.

### 3.3 Common miRNAs Regulating Target Genes

We employed a multi‐database approach to identify potential miRNA regulators of IL‐12A, IL‐12B, IL‐15, IL‐18, and IFNG genes. We selected miRNAs predicted by a minimum of three databases and further chose those targeting at least two of the five genes. This analysis revealed a pattern of single miRNAs regulating multiple genes. Notably, hsa‐miR‐590‐3p, hsa‐miR‐380‐3p, hsa‐miR‐340‐5p, and hsa‐miR‐495‐3p exhibited the most extensive regulatory potential, targeting four and three genes, respectively (Table 1).

**TABLE 1:** List of common miRNAs for IL‐12A, IL‐12B, IL‐15, IL‐18, and IFNG genes.

	IL‐12A	IL‐12B	IL‐15	IL‐18	IFNG
hsa‐miR‐5692a	*	*			*
hsa‐miR‐607	*				*
hsa‐miR‐380‐3p	*	*			
hsa‐miR‐340‐5p	*		*		*
hsa‐miR‐664a‐3p	*				*
hsa‐miR‐23a‐3p		*	*		
hsa‐miR‐23b‐3p		*	*		
hsa‐miR‐590‐3p		*	*	*	*
hsa‐miR‐6755‐3p		*			*
hsa‐miR‐4284		*			*
hsa‐miR‐205‐5p		*	*		
hsa‐miR‐29a‐3p		*			*
hsa‐miR‐29b‐3p		*			*
hsa‐miR‐29c‐3p		*			*
hsa‐miR‐495‐3p		*	*		*
hsa‐miR‐513a‐5p		*			*
hsa‐miR‐587		*			*
hsa‐miR‐891b		*	*		
hsa‐miR‐132‐3p		*	*		
hsa‐miR‐212‐3p		*	*		
hsa‐miR‐27a‐3p		*			*
hsa‐miR‐27b‐3p		*			*
hsa‐miR‐450b‐5p		*	*		
hsa‐miR‐507		*	*		
hsa‐miR‐513a‐3p		*	*		
hsa‐miR‐143‐3p				*	*
hsa‐miR‐526b‐5p			*	*	
hsa‐miR‐130a‐3p			*	*	
hsa‐miR‐130b‐3p			*	*	
hsa‐miR‐301a‐3p			*	*	
hsa‐miR‐301b‐3p			*	*	
hsa‐miR‐454‐3p			*	*	
hsa‐miR‐181a‐5p			*		*
hsa‐miR‐181b‐5p			*		*
hsa‐miR‐181c‐5p			*		*
hsa‐miR‐181d‐5p			*		*
hsa‐miR‐369‐3p			*		*
hsa‐miR‐579‐3p			*		*

*Note:* “*” in the table means that each gene is targeted by which microRNA.

### 3.4 Homology Analysis and miRNAs Interaction via Targeted Genes

**FIGURE 3:**
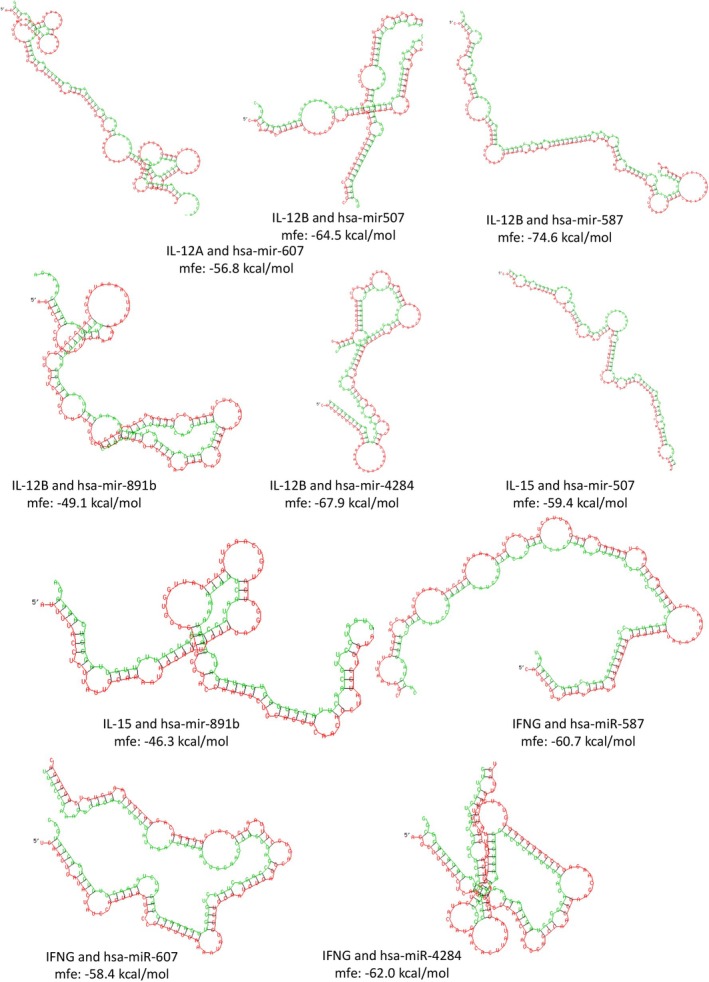
miRNA (green) versus mRNA (red) pairing using RNAhybrid algorithm.

Homology analysis of IL‐12A, IL‐12B, IL‐15, IL‐18, and IFNG genes with target miRNAs homology analysis determined that most duplexes had MFE values below the threshold (−15 kcal/mol). Moreover, hsa‐miR‐607 with IL‐12A, hsa‐miR‐587, hsa‐miR‐4284, hsa‐miR‐507, and hsa‐miR‐891b with IL‐12B, hsa‐miR‐507, and hsa‐miR‐891b with IL‐15, hsa‐miR‐4284, hsa‐miR‐587, and hsa‐miR‐607 with IFNG all exhibited possible interactions with MFE values ≤ −30 (Figure 3).

### 3.5 Construction of the miRNA–mRNA Regulatory Network

**FIGURE 4:**
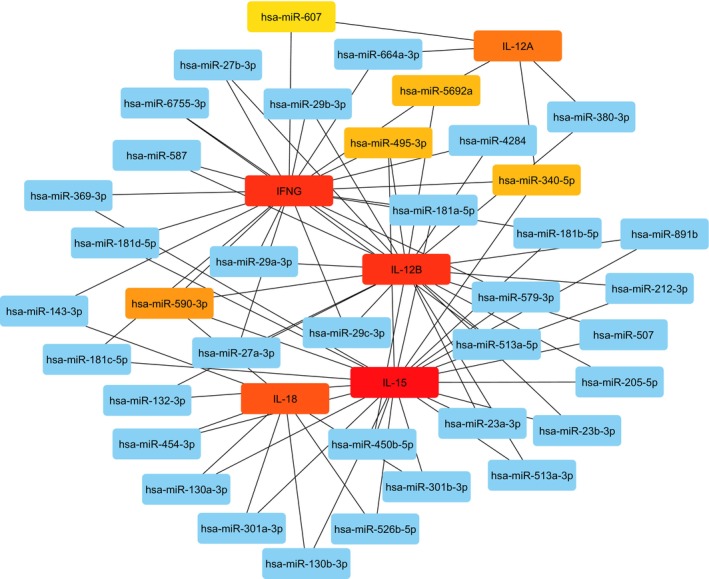
The miRNA‐mRNA regulatory network of IL‐12A, IL‐12B, IL‐15, IL‐18, and IFNG genes and the targeted miRNAs.

A miRNA‐mRNA regulatory network was constructed to visualize the interactions between five cytokine genes (IL‐12A, IL‐12B, IL‐15, IL‐18, and IFNG) and 38 miRNAs, focusing on miRNAs targeting multiple cytokine genes. This network sheds light on the potential role of these miRNAs in NK cell suppression. Notably, the analysis using CytoHubba identified 10 key nodes within the network: IL‐15, IFNG, IL‐12B, IL‐18, and IL‐12A, along with five miRNAs—hsa‐miR‐590‐3p (with the most interactions), hsa‐miR‐340‐5p, hsa‐miR‐495‐3p, hsa‐miR‐5692a, and hsa‐miR‐130a‐3p (Figure 4).

### 3.6 GO and KEGG Pathway Analysis

**FIGURE 5:**
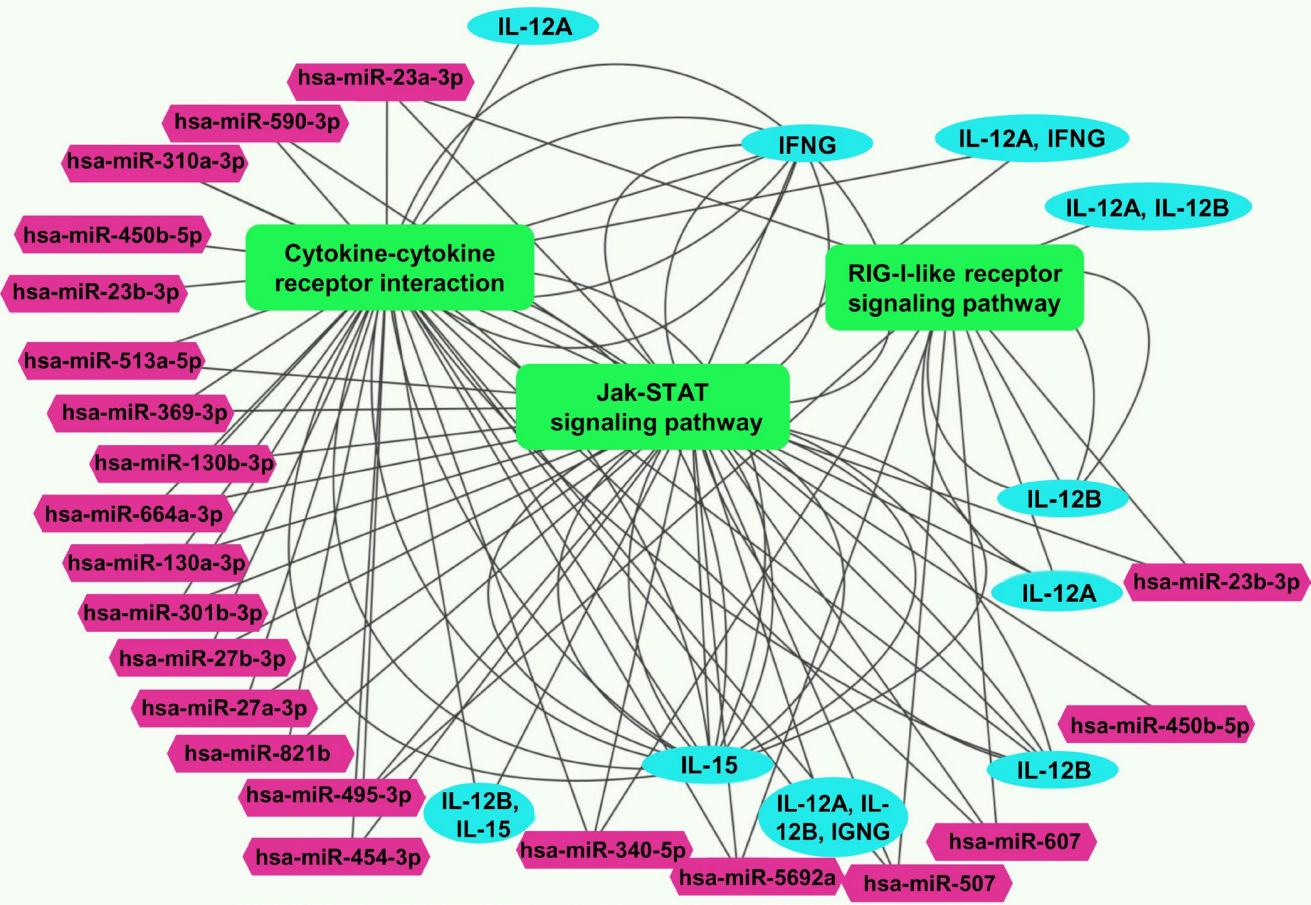
miRNA‐mRNA interaction network analysis and KEGG pathways enrichment.

**FIGURE 6:**
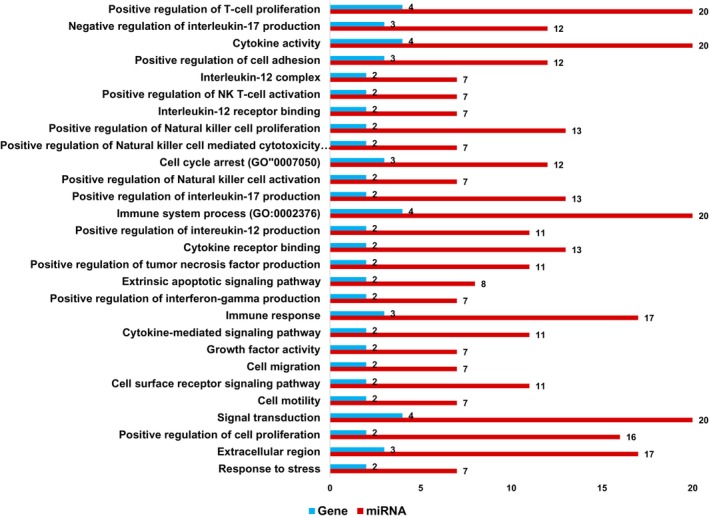
GO enrichment analysis of target miRNA of IL‐12A, IL‐12B, IL‐15, and IFNG genes predicted by microT‐CDS, and TargetScan.

**FIGURE 7:**
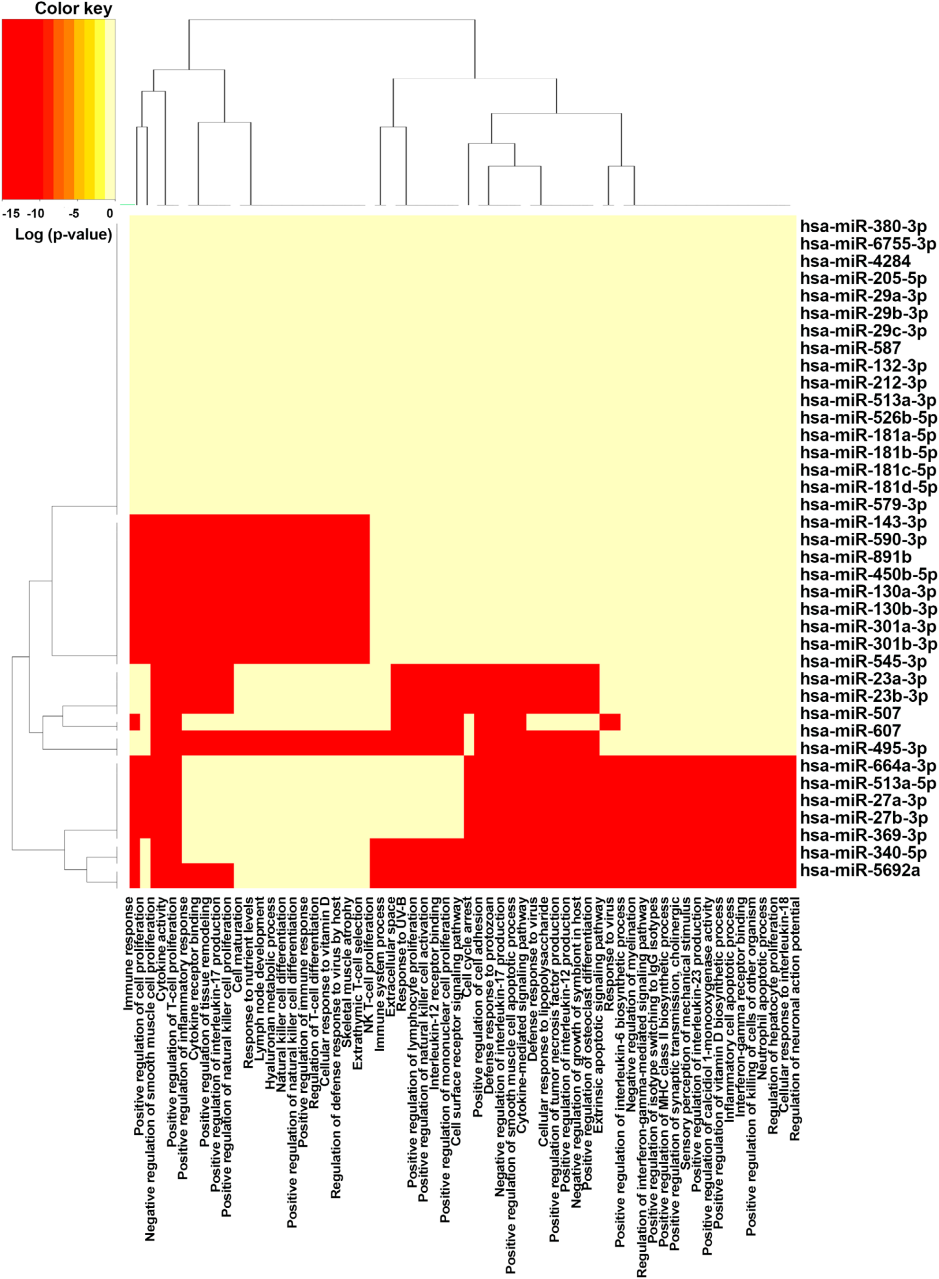
The heatmap illustrates the enrichment of signaling pathways targeted by the identified miRNAs. The *x*‐axis represents the enriched pathways, while the y‐axis lists the miRNAs. The hierarchical clustering applied to both axes groups the miRNAs and pathways based on similarity in enrichment patterns. Red regions represent pathways with highly significant enrichment (lower *p*‐values). Pale yellow regions represent pathways with low significance enrichment for that miRNA‐pathway pair.

To understand the functional implications of the identified miRNA‐gene interactions, we performed pathway enrichment analysis using DIANA‐miRPath v.3 software. This analysis identified three KEGG pathways with statistically significant enrichment (*p*‐value < 0.05) that are known to play a role in various cancers (Table 2 and Figure 5). Additionally, gene ontology (GO) analysis revealed enrichment for specific GO categories associated with the differentially expressed genes and miRNAs. Accordingly, we excluded terms with a single gene count as they lack sufficient interpretability. The filtered results present enriched GO terms that are supported by at least two genes (Figure 6). Notably, the heatmap visualization suggests a potential role for some of these miRNAs in NK cell and cancer‐related pathways (Figure 7). The heatmap colors represent the statistical significance of pathway enrichment, with colors corresponding to the log‐transformed *p*‐values (−log10 *p*‐value). The red regions indicate highly significant associations (lower *p*‐values), while the pale yellow regions represent less significant associations (higher *p*‐values).

**TABLE 2:** Top 3 KEGG pathways enriched by target miRNA of IL‐12A, IL‐12B, IL‐15, and IFNG genes predicted by microT‐CDS, TarBase, and TargetScan.

MicroT‐CDS prediction	MiRNA	*p*	Gene count
KEGG pathway			
Jak–STAT signaling pathway	Number = 20	0.0028	Number = 4
	hsa‐miR‐5692a		IL‐12A, IL‐12B, IFNG
	hsa‐miR‐607		IL‐12A
	hsa‐miR‐340‐5p		IL‐12A, IFNG
	hsa‐miR‐664a‐3p		IFNG
	hsa‐miR‐513a‐5p		IFNG
	hsa‐miR‐27a‐3p		IFNG
	hsa‐miR‐27b‐3p		IFNG
	hsa‐miR‐369‐3p		IFNG
	hsa‐miR‐23a‐3p		IL12B
	hsa‐miR‐23b‐3p		IL12B
	hsa‐miR‐495‐3p		IL12B, IL15
	hsa‐miR‐507		IL12B
	hsa‐miR‐590‐3p		IL15
	hsa‐miR‐891b		IL15
	hsa‐miR‐450b‐5p		IL15
	hsa‐miR‐130a‐3p		IL15
	hsa‐miR‐130b‐3p		IL15
	hsa‐miR‐301a‐3p		IL15
	hsa‐miR‐301b‐3p		IL15
	hsa‐miR‐454‐3p		IL15
Cytokine‐cytokine receptor interaction	Number = 20	0.019	Number = 4
	hsa‐miR‐5692a		IL‐12A, IL‐12B, IFNG
	hsa‐miR‐23a‐3p		IL12B
	hsa‐miR‐23b‐3		IL12B
	hsa‐miR‐495‐3p		IL12B, IL15
	hsa‐miR‐507		IL12B
	hsa‐miR‐607		IL12A
	hsa‐miR‐340‐5p		IL‐12A, IFNG
	hsa‐miR‐590‐3p		IL15
	hsa‐miR‐891b		IL15
	hsa‐miR‐450b‐5		IL15
	hsa‐miR‐130a‐3p		IL15
	hsa‐miR‐130b‐3p		IL15
	hsa‐miR‐301a‐3p		IL15
	hsa‐miR‐301b‐3p		IL15
	hsa‐miR‐454‐3p		IL15
	hsa‐miR‐664a‐3p		IFNG
	hsa‐miR‐513a‐5p		IFNG
	hsa‐miR‐27a‐3p		IFNG
	hsa‐miR‐27b‐3p		IFNG
	hsa‐miR‐369‐3p		IFNG
RIG‐I‐like receptor signaling pathway	Number = 7	0.026	Number = 2
	hsa‐miR‐5692a		IL‐12A, IL‐12B
	hsa‐miR‐607		IL‐12A
	hsa‐miR‐340‐5p		IL‐12A
	hsa‐miR‐23a‐3p		IL‐12B
	hsa‐miR‐23b‐3p		IL‐12B
	hsa‐miR‐495‐3p		IL‐12B
	hsa‐miR‐507		IL‐12B

## 4 Discussion

Considerable improvement has been made in comprehending the initiation, spread, and the immune system's function in malignancies. The immune system plays a critical role in preventing cancer cell dissemination and metastasis to other organs. Notably, NK and T cells are the most common immune effector cells utilized in various cancer immunotherapies to enhance the anti‐tumor response [20, 21, 22].

Cytokines are fundamental for NK cell activation and maintenance, acting either independently or synergistically. For example, IL‐21 enhances human NK cell cytotoxicity, IFNG production, and antibody‐dependent cellular cytotoxicity (ADCC) responses. Furthermore, IL‐21 can repress the proliferation and induce apoptosis of NK cells. However, when combined with IL‐2 or IL‐15, IL‐21 exhibits a synergistic activation effect [20, 23].

Studies demonstrate that NK cells pre‐activated with a combination of IL‐12, IL‐15, and IL‐18 exhibit enhanced and sustained antitumor activity following infusion in both in vitro and in vivo models. These pre‐activated NK cells display characteristics of “memory‐like” cells, demonstrating a heightened response to subsequent cytokine stimulation or reactivation through activating receptors [24]. IL‐18 has a dual effect on NK cells. It enhances IFNG production, which can be both beneficial and detrimental. While high IFNG levels can reduce NK cell cytotoxicity, this cytokine also correlates with increased anti‐tumor immunity [25].

This study investigated the relationship between NK cell infiltration and cytokine expression in various cancers. We found a positive correlation between NK cell infiltration and the expression of IL‐12, IL‐15, IL‐18, and IFNG, highlighting the potential interplay between these factors in the anti‐tumor response. Furthermore, a protein–protein interaction network was created for biological investigation. Our findings indicated significant interactions, suggesting that these proteins constitute a biologically associated group.

The cytokines protein–protein interaction network for illustrating potential molecules in rheumatoid arthritis was explored. They employed PPI and gene expression data to create a two‐dimensional informational framework for their methodology. Their results demonstrated protein–protein interactions in cytokine signaling pathways. This network‐based approach, which helped uncover important molecules in the cytokine signaling network, may be utilized to identify several biomarkers to monitor treatment effectiveness [26].

Our findings propose that the IL‐12A/B, IL‐15, IL‐18, and IFNG gene network plays a critical role in tumor development and treatment. Pathway analysis revealed their involvement in essential cellular processes, including cytokine‐cytokine receptor interaction, JAK–STAT signaling, pathways in cancer, and Th1/Th2 cell differentiation. This interconnected network highlights the potential for targeting these genes or their pathways to improve cancer therapies.

Patidar et al. emphasized the crucial role of damage‐associated molecular patterns (DAMPs) and toll‐like receptors (TLRs) in affecting JAK–STAT pathways that modulate immune responses, particularly through cytokine release that influences T‐cell activation and tumor response [27]. This aligns with our findings, which identify the IL‐12A/B, IL‐15, IL‐18, and IFNG gene networks as pivotal in tumor progression and treatment. Both studies underscore the importance of cytokine interaction, while the DAMP‐TLR axis orchestrates the immune environment and alters T‐cell dynamics. Our research highlights the JAK–STAT signaling pathways as essential mediators in these processes. The interplay between these pathways suggests that targeting the cytokine networks involved in TLR signaling could enhance therapeutic strategies, potentially leading to more effective cancer treatments. Thus, integrating insights from both studies could inform a comprehensive approach to immunotherapy, focusing on the regulatory mechanisms that govern TME interactions and immune cell functionalities.

NK cells possess a diverse array of cell surface receptors that recognize cytokines like IL‐12, IL‐15, IL‐18, and IFNG. Ligand binding triggers the JAK–STAT signaling pathway, activating NK cells. This activation can result in both cytokine production, such as IFNG, and the elimination of target cells [28]. Cytokine stimulation activates NK cells via the JAK–STAT signaling pathway. JAK kinases phosphorylate STAT transcription factors, triggering their translocation to the nucleus. There, activated STATs induce the expression of genes critical for NK cell activation and effector functions [29]. NK cells are important in the anti‐tumor immune process. They directly eliminate tumor cells and secrete cytokines that activate other immune cells. Notably, IL‐12 and IFNG facilitate the differentiation of Th cells towards the Th1 subtype, which are more potent at eliminating tumors [5]. Th cells can differentiate into two subsets: Th1 and Th2, each with distinct roles in the immune response. Th1 cells are geared towards fighting intracellular pathogens and cancer through the production of IFNG. Conversely, Th2 cells promote allergic responses by secreting IL‐4. A healthy immune system relies on a balanced interplay between Th1 and Th2 activity [30].

NK cells are crucial for the anti‐tumor immune response. Activated by cytokines through the JAK–STAT pathway, they produce cytokines like IFNG and directly eliminate target cells. Additionally, NK cells promote the differentiation of Th1 cells, which are more effective at combating cancer. A strong immune system depends on a balanced interaction between Th1 and Th2 activity [31].

Our findings demonstrate a positive correlation between IL‐12A/B, IL‐15, IL‐18, and IFNG expression and NK cell infiltration across various cancers. This aligns with prior research highlighting the role of these cytokines in NK cell activation and recruitment to tumors. Notably, the strong association between IL‐15 and IFNG levels with NK cell infiltration is particularly intriguing. These cytokines are well‐established as potent NK cell activators, and their production often increases in cancer patients. This suggests a potential role for NK cells in early tumor development and suppression. Supporting this notion, a study by Gillgrass et al. observed significantly higher IL‐12A and IL‐15 expression in NK cells from breast cancer patients compared to healthy controls. Furthermore, their study mirrored our results, demonstrating a positive correlation between these cytokine levels and NK cell infiltration at tumor sites [32]. However, several studies highlight potential limitations to NK cell function in cancer. Niu et al. observed decreased cytotoxicity and IFNG secretion by peripheral blood NK cells from lung cancer cases compared to normal controls. Similarly, another study reported a correlation between faster NK cell expansion in cancer patients and a reduction in their cytotoxic and IFNG production capacities. These findings suggest that while NK cells may infiltrate tumors, their functionality can be compromised in the tumor microenvironment, potentially hindering their anti‐tumor potential [33, 34].

NK cells exhibit distinct transcriptional profiles as they differentiate into various functional subsets. These subsets play crucial roles in immune responses. Emerging research highlights the importance of miRNAs in NK cells. These small regulatory molecules have been implicated in diverse NK cell processes, including activation, maturation, development, cytotoxicity, proliferation, and cytokine production [35, 36].

Prior studies have explored miRNA function in mature murine NK cells by deleting Dicer or Dgcr8, key enzymes in miRNA biogenesis. These experiments showed that miRNAs play a critical role in cell survival, with deficiencies in Dicer and Dgcr8 leading to increased NK cell death [37]. Furthermore, the absence of miRNAs impaired responsiveness through cytokine receptors, suggesting a role in NK cell signaling [38].

Stimulation with IL‐12 and IL‐18 triggers a significant increase in miR‐155 expression within NK cells compared to unstimulated cells. In human NK cells, this miRNA acts as a positive regulator of IFNG production. Similar effects were observed with miR‐362‐5p overexpression, further highlighting the role of miRNAs in stimulating IFNG production. These findings collectively suggest that miRNAs contribute to the cytotoxic potential of NK cells by regulating the production of inflammatory cytokines [39].

Following our analysis of cytokines and their link to NK cells, we employed various databases to identify and predict potential miRNA regulators of these genes. Interestingly, the results revealed that single miRNAs can target multiple genes involved in both innate and adaptive immunity. This observation aligns with existing knowledge demonstrating the pleiotropic nature of miRNAs, which can influence diverse cellular processes [40]. We also noted that some miRNAs were more commonly shared between the target genes than others. For example, hsa‐miR‐590‐3p is shared between all five target genes including IL‐12A, IL‐15, IL‐18, and IFNG; hsa‐miR‐380‐3p and hsa‐miR‐340‐5p are shared among four target genes, and hsa‐miR‐495‐3p is shared between three target genes. Moreover, some miRNAs are found in more databases than others. For instance, hsa‐miR‐590‐3p appears in all five databases, while hsa‐miR‐607 is only found in one database. This suggests that hsa‐miR‐590‐3p may be more important for regulating the immune response than hsa‐miR‐607. The findings from this analysis have significant implications for understanding the regulation of the immune response. We propose that miRNAs play a crucial role in coordinating the immune response by regulating multiple genes involved in this process. Further analysis is needed to investigate the specific mechanisms by which miRNAs regulate the immune response and to determine the clinical relevance of these findings.

Mirroring the miRNA sharing patterns, our homology analysis revealed stable duplex formation between certain target genes and miRNAs. This stability, indicated by MFE values less than or equal to −30 kcal/mol for miRNAs like hsa‐miR‐607, hsa‐miR‐587, hsa‐miR‐4284, hsa‐miR‐507, and hsa‐miR‐891b, suggests a high likelihood of interaction and potential for these miRNAs to effectively regulate their target genes.

The constructed miRNA‐mRNA regulatory network sheds light on the complex interplay between miRNAs and cytokines governing NK cell activity. Notably, hsa‐miR‐590‐3p emerged as the most connected miRNA, interacting with a higher number of cytokine genes compared to hsa‐miR‐340‐5p, hsa‐miR‐495‐3p, hsa‐miR‐5692a, and hsa‐miR‐130a‐3p. This suggests a potentially more prominent role for hsa‐miR‐590‐3p in regulating cytokine expression and influencing NK cell suppression.

Our findings align with prior research demonstrating miRNA‐mediated regulation of cytokine expression [41]. While this study uncovers potential regulatory interactions between identified miRNAs and cytokines, further investigations are necessary to validate these interactions experimentally and elucidate their functional impact on NK cell activity.

Gene set enrichment analysis identified significant GO pathways and KEGG signaling pathways (*p*‐value < 0.05). The top three enriched KEGG pathways for target miRNAs of IL‐12A/B, IL‐15, and IFNG genes were the Jak–STAT signaling pathway, cytokine–cytokine receptor interaction, and RIG‐I‐like receptor signaling. These pathways all play critical roles in immune regulation, suggesting miRNA involvement in their modulation. Notably, the Jak–STAT pathway, a cornerstone of both innate and adaptive immunity, is activated by cytokine‐receptor binding and drives the transcription of genes crucial for immune cell activation and differentiation [42]. The enriched pathways further highlight miRNA involvement in regulating key immune processes. The cytokine–cytokine receptor interaction pathway, previously mentioned, underscores miRNA's role in immune signaling. Additionally, the RIG‐I‐like receptor signaling pathway, crucial for innate immunity, suggests miRNA modulation of viral recognition and immune cell activation [43]. Our analysis revealed enrichment of key immune signaling pathways, suggesting miRNA involvement in their regulation. This finding holds promise for novel cancer immunotherapy strategies. By targeting these genes and their associated miRNAs, we could potentially enhance NK cell function and improve treatment efficacy. This might involve utilizing miRNA‐targeting agents or gene therapy to manipulate these regulatory elements.

Accordingly, validation through laboratory experiments and larger clinical trials is essential to confirm these findings. Additionally, it is important to clarify how these genes and miRNAs suppress NK cell function, as this represents a key area for future research. Future studies should also focus on experimentally validating the identified miRNA targets to further establish their role in regulating NK cell activity. Gaining a deeper understanding of these mechanisms will be crucial for unraveling their involvement in cancer and developing more effective immunotherapy strategies.

## 5 Conclusion

In conclusion, this study demonstrated that the IL‐12A, IL‐12B, IL‐15, IL‐18, and IFNG genes, along with their target miRNAs, are significantly associated with NK cell infiltration and suppression in various types of cancer. Our findings suggest that these genes and their targeted miRNAs may serve as potential factors for monitoring NK cells and developing therapeutic strategies for cancer immunotherapy. This could involve the use of miRNA‐targeting agents or gene therapy to enhance the expression of these genes.

## Author Contributions

A.Z.T. contributed to data collection and writing and drafting of the manuscript. S.V. participated in data collection and analysis, as well as manuscript writing, ensuring clarity, and coherence throughout the document. A.A.S. provided scientific and technical oversight, managing the revision process to enhance the manuscript's quality. All authors comprehensively reviewed the manuscript and approved the final revised version.

## Ethics Statement

The authors have nothing to report.

## Conflicts of Interest

The authors declare no conflicts of interest.

## Data Availability

Data sharing is not applicable to this article as no new data were created or analyzed in this study.
